# Loneliness and Depression Among Female University Students During the COVID-19 Pandemic: A Cross-Sectional Study in Riyadh, Saudi Arabia, 2020

**DOI:** 10.3389/ijph.2022.1604885

**Published:** 2022-08-24

**Authors:** Deemah Alateeq, Alhanouf Aljabri, Ghada Aldogiam, Haya Alajmi, Hussah Alsoqaih, Rawan Alfadhly, Raneem Alshahrani

**Affiliations:** Clinical Sciences Department, College of Medicine, Princess Nourah bint Abdulrahman University, Riyadh, Saudi Arabia

**Keywords:** loneliness, students, COVID-19, depression, females

## Abstract

**Objectives:** Quarantine-related loneliness has impacted university students during the coronavirus disease 2019 (COVID-19) pandemic. This study aims to evaluate loneliness among female university students in Riyadh, Saudi Arabia during the pandemic and to investigate its correlation with depression.

**Methods:** A sample of 753 female students was collected during the first COVID-19 summer vacation outbreak through a cross-sectional survey that consisted of two parts: 1) Sociodemographic characteristics and COVID-19 related questions; 2) The eight-item UCLA Loneliness Scale (ULS-8) and the Patient Health Questionnaire-9 (PHQ-9).

**Results:** Most participants were between 18 and 22 years old (75.2%) and were studying at humanities college (61.50%). Loneliness and depression were reported among 63.3% and 41.1% of female students, respectively, and the correlation between them was significantly positive (beta = 0.419, *p* < 0.001). Having a previous history of a psychiatric condition and families with insufficient monthly income were the main associated factors with high levels of loneliness and depression.

**Conclusion:** Female university students experienced loneliness and depression under quarantine during the first COVID-19 summer vacation outbreak. Psychosocial intervention for the vulnerable groups is essential, as are longitudinal studies.

## Introduction

Coronavirus disease 2019 (COVID-19) is a novel communicable viral respiratory disease caused by new strains of severe acute respiratory syndrome coronavirus (SARS-CoV-2) [1]. The World Health Organization (WHO) declared the COVID-19 outbreak a public health emergency of international concern (PHEIC) on 30 January 2020, and with the virus spreading worldwide, WHO declared the COVID-19 outbreak could be characterized as a pandemic on 11 March [2]. The first case was reported in Saudi Arabia on 7 March, and increasing numbers were seen all over the world with increased physical distancing and many preventive precautions, including shifting the education system online [3, 4].

COVID-19 doesn’t affect only physical health; it also has a serious negative impact on the psychological well-being of the general population, especially among younger people and females. Many recent Saudi studies have reported significant levels of depression, anxiety, stress, and obsessive-compulsive symptoms among the general population during the pandemic [5–8]. When comparing mental health with pre-COVID-19 in Saudi Arabia, the risk of depression and anxiety was relatively high among general population [9]. Another study showed that the mental health of about one-third of university students in Saudi Arabia were worsened during the pandemic, including anxiety, depression, stress and sleep [10]. And those who were isolating themselves had higher levels of anxiety, depression, and stress [10]. Moreover, in March 2020, as education moved online during the early stage of the pandemic in Saudi Arabia, moderate to high levels of perceived stress were reported among 85% of participating students with a range of educational levels. However, it was significantly higher among university and female students [11]. And in April 2020, two studies were conducted in Saudi Arabia to assess the mental health impact of the pandemic on university students [12, 13]. Symptoms of anxiety, depression, and moderate to high stress were reported among Saudi university students (40.8%, 48.8%, and 86.7%, respectively). These symptoms were significantly higher among females and younger individuals [12]. In addition, symptoms of post-traumatic stress disorder were experienced by 30.9% of Saudi university students; the symptoms were significantly higher in females [13].

Loneliness can arise from a thwarted ability to meet the needs of belongingness and social connection. During the COVID-19 pandemic, students lost interaction, lacked emotional support, and experienced social isolation. These fundamental changes are associated with loneliness, depression, and suicidal thoughts [14, 15]. To our knowledge, no study has evaluated loneliness among university students in Saudi Arabia during the pandemic. This study evaluated quarantine-related loneliness among female university students during the COVID-19 pandemic in Saudi Arabia and investigated its correlation with depression and demographic variables.

## Methods

### Study Population and Sample

The participants of this study were female undergraduate students who were studying at Princess Nourah bint Abdulrahman University (PNU) or King Saud University (KSU), which are the main universities in Riyadh, Saudi Arabia. The cross-sectional survey was distributed to the participants through their university emails and students’ What’s Up app groups during the first COVID-19 outbreak during the summer vacation of July and August 2020. Data was collected using the convenience sampling technique, which was carried out using an online platform link. Participants received a message from students’ representatives at each college that include the link of the questionnaire, the time needed to fill the survey, and the aim of the study. Consent was required from all participants before they could start filling the survey. Ethical approval was obtained from the Institutional Review Board at PNU (IRB-PNU:20-0225) in Riyadh, Saudi Arabia.

### Data Collection Tools

A self-reported questionnaire was designed to collect the data required for the purposes of the study. Part one of the questionnaire is germane to the participants’ demographic specifications, including age, nationality, marital status, region of residence, family income, college, level of study, GPA (The Grade Point Average), and health conditions, in addition to some items about the quarantine as well as their history and their families’ history with COVID-19. Part two of the questionnaire measures loneliness and depression using Arabic versions of the eight-item University of California, Los Angeles (UCLA) Loneliness Scale (ULS-8) and Patient Health Questionnaire 9 (PHQ-9). The UCLA loneliness scale is used to screen for loneliness [16]. The short version is used widely and includes eight items [17]. Each item is rated on a four-point Likert scale ranging from 1 (never) to 4 (always). However, in the Arabic version of the scale, seven items have been assessed as adequate for measuring loneliness, with a total score ranging between 7 and 28 points. Higher scores indicate higher levels of loneliness. The PHQ-9 was then used to screen for depression using nine items. The PHQ-9 is also a four-point Likert scale ranging from 0 (not at all) to 3 (almost every day), with a total score ranging from 0 to 27 points. Higher scores indicate higher severity of depression. The Arabic versions of both scales are valid and reliable [18, 19].

### Statistical Analysis

Significant differences between variables in loneliness and depression scores were explored using a Student’s t-test, chi-square test, or one-way ANOVA test. Correlations between scale scores were investigated using Spearman’s coefficient. A 0.05 level of significance was used. A linear regression model was used to determine how the loneliness and depression scores can be predicted from significantly associated variables. All analyses were made using the functions library of the Statistical Product and Service Solutions 20.0 (SPSS 20.0).

## Results

### Sociodemographic and COVID-19 Related Characteristics

A total of 753 female university students enrolled themselves electively into the study from two universities in Riyadh city, the capital of Saudi Arabia. The sociodemographic and COVID-19 related characteristics of the students are shown in Table 1. Most of the students were aged between 18 and 22 years (75.2%), were not married (92.7%), did not have children (96%), were in Riyadh during the vacation (93.9%), had sufficient monthly income (77.4%), and were students at colleges of humanities (61.5%) with a GPA above 4 out of 5 (73.6%). Almost half were junior students (45.6%). Only 9.7% and 10.5% of students had been diagnosed with medical and psychiatric conditions, respectively. Regarding COVID-19 related questions, most of the students complied with home quarantine (72.2%) and spent 60 days or more in quarantine (74%). Moreover, only 1.9% of them have been diagnosed with COVID-19 disease, but 45.8% of them had a family or friend who had been diagnosed with COVID-19 disease.

**TABLE 1:** Sociodemographic and coronavirus disease 2019 pandemic related characteristics, loneliness, and depression (N = 753) (Riyadh, Saudi Arabia. 2020).

Variables	Total	ULS-8 (Out of 28)		PHQ-9 (Out of 27)	
	N (%)	Mean (SD)	P value	Mean (SD)	P value
Age group					
18–22 years	566 (75.2)	17.64 (4.89)	0.472	11.063 (6.32)	0.835
23–26 years or older	187 (24.8)	17.93 (4.71)		11.18 (6.78)	
Marital state					
Never married	698 (92.7)	17.75 (4.93)	0.386	11.10 (6.52)	0.896
Ever married	55 (7.3)	17.16 (3.65)		10.98 (5.32)	
Children					
No	723 (96)	17.74 (4.88)	0.351	11.14 (6.47)	0.329
Yes	30 (4)	16.90 (3.86)		9.967 (5.38)	
Region of residence					
Riyadh	707 (93.9)	17.10 (4.01)	0.346	9.74 (5.03)	0.069
Other regions	46 (6.1)	17.75 (4.89)		11.18 (6.51)	
Family’s monthly income				11.063 (6.32)	
In debt	17 (2.3)	19.76 (5.13)	<0.001	15.10 (8.40)	<0.001
Not enough	153 (20.3)	20.16 (4.3)		14.73 (6.36)	
Just enough	367 (48.7)	16.78 (4.92)		10.01 (6.10)	
Enough with some savings	216 (28.7)	17.38 (4.40)		10.04 (5.85)	
College					
College of humanities	463 (61.5)	17.43 (4.91)	0.087	11.10 (6.45)	0.138
College of health science	100 (13.3)	17.67 (4.98)		10.10 (6.81)	
College of science	104 (13.8)	17.75 (4.86)		12.32 (6.90)	
College of education	86 (11.4)	18.01 (4.18)		11.0 (5.10)	
Study Level					
Junior	343 (45.6)	17.61 (4.84)	0.843	11.22 (6.65)	0.870
Sophomore	309 (41)	17.83 (4.88)		11.01 (6.10)	
Senior	101 (13.3)	17.66 (4.80)		10.90 (6.74)	
GPA					
≤4 points out of 5	199 (26.4)	17.93 (5.13)	0.435	11.85 (6.83)	0.053
>4 points out of 5	554 (73.6)	17.62 (4.75)		10.82 (6.27)	
Diagnosed with medical condition					
No	680 (90.3)	17.62 (4.90)	0.075	10.98 (6.45)	0.150
Yes	73 (9.7)	18.58 (4.27)		120.12 (6.24)	
Diagnosed with psychiatric condition					
No	674 (89.5)	17.40 (4.80)	<0.001	10.54 (6.15)	<0.001
Yes	79 (10.5)	20.37 (4.48)		15.79 (6.91)	
Complied with home quarantine					
No	209 (27.8)	17.48 (4.62)	0.419	10.67 (5.94)	0.265
Yes	544 (72.2)	17.80 (4.94)		11.25 (6.61)	
Days spent in quarantine					
20–39 days	66 (8.8)	16.86 (4.83)	0.330	9.66 (6.39)	0.017
40–59 days	130 (17.3)	17.72 (4.64)		10.13 (5.77)	
60 days or more	557 (74)	17.81 (4.89)		11.48 (6.55)	
Diagnosed with COVID-19 disease					
No	739 (98.1)	17.74 (4.88)	0.009	11.11 (6.45)	0.495
Yes	14 (1.9)	15.86 (2.25)		9.93 (4.70)	
Family or friend diagnosed currently with COVID-19					
No	408 (54.2)	17.85 (5.11)	0.401	11.22 (6.47)	0.543
Yes	345 (45.8)	17.55 (4.52)		10.93 (6.40)	

### Loneliness and Depression

**FIGURE 1:**
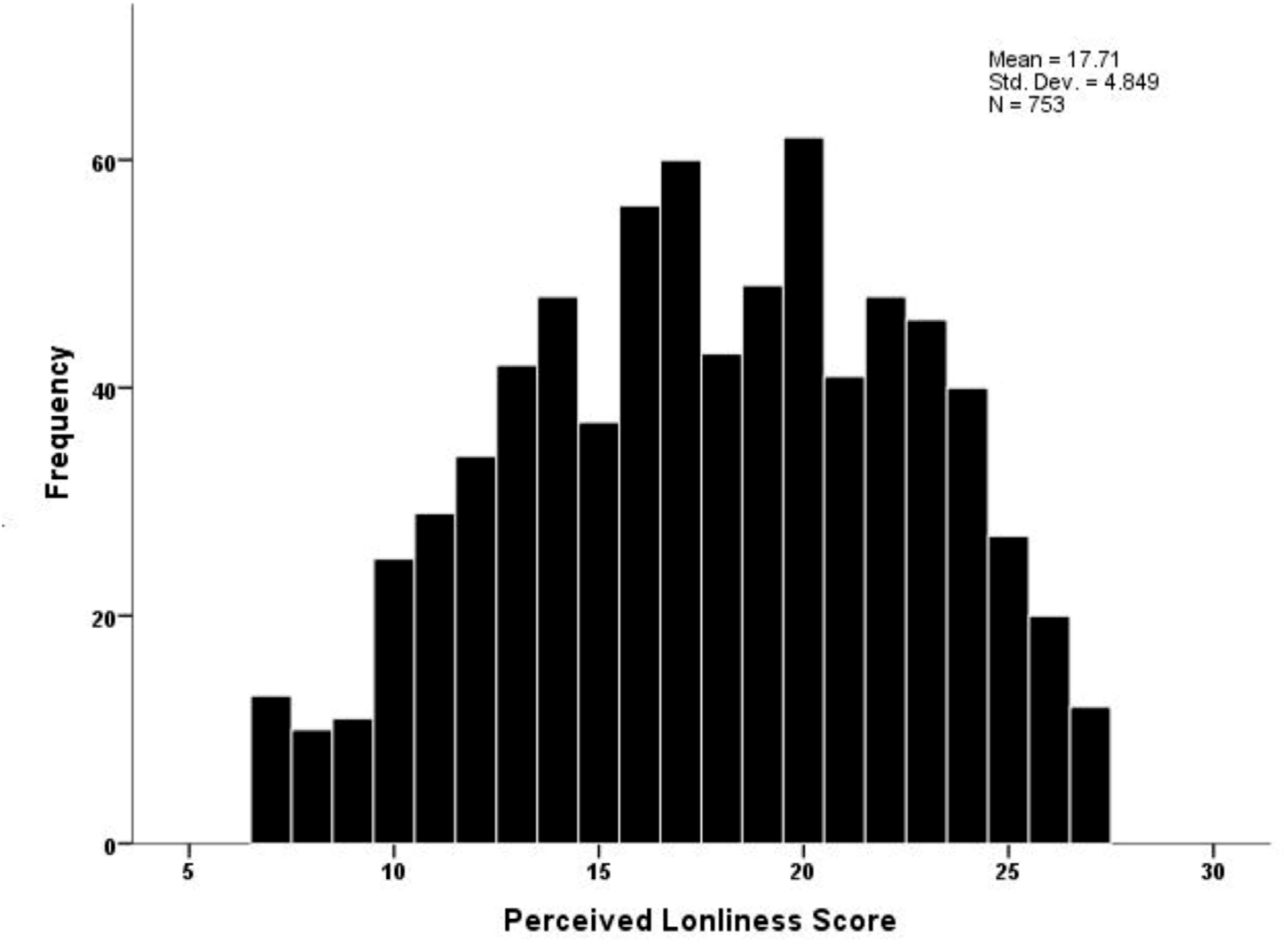
A histogram for the distribution of loneliness mean score among female students (Riyadh, Saudi Arabia. 2020).

**FIGURE 2:**
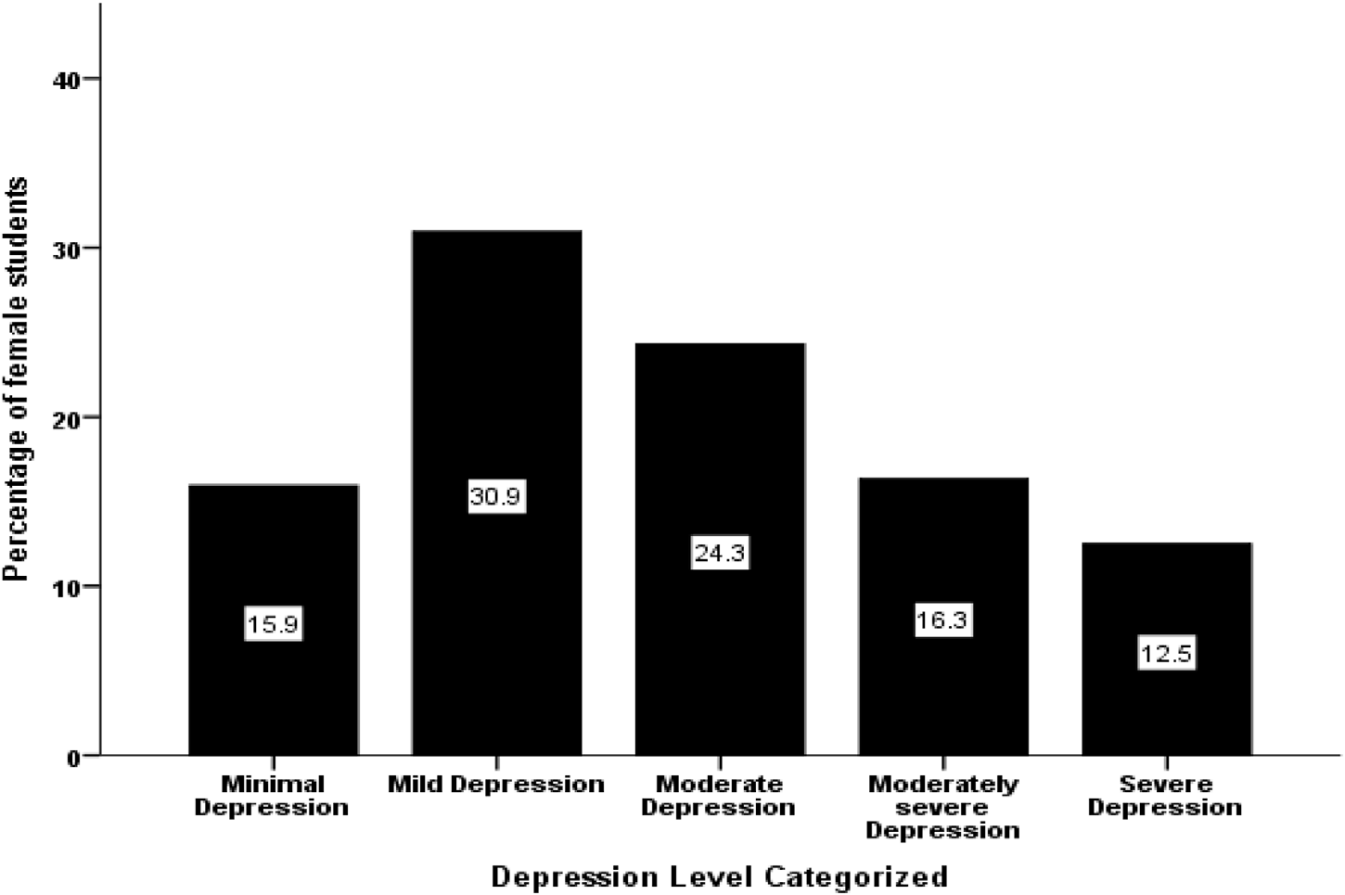
Levels of depression score among female students (Riyadh, Saudi Arabia. 2020).

Female students’ loneliness mean was 17.71 (4.84) points out of a maximum 28 points on the ULS-7, which, expressed as a percentage, equals 63.3%. Figure 1 displays loneliness score among the participants. Also, female students’ depression mean was 11.10 (6.44) points out of maximum 27 points on the PHQ-9, or 41.1%. Figure 2 displays depression levels among the sample varied from mild (30.9%) to moderate (24.3%) to moderately high (16.3%) to severe (12.5%). The bivariate Pearson correlation (r) test showed that the female students’ loneliness scores correlated significantly and positively with their depression scores (*r* = 0.679, *p* < 0.010).

Table 1 displays the bivariate correlations for the female students’ perceived sense of loneliness and depression. Most of the sociodemographic characteristics did not correlate significantly with either the loneliness or depression mean. Students with a positive history of a medical condition had a slightly higher level of loneliness (M = 18.58, SD = 4.27) compared to those with a negative medical history (M = 17.62, SD = 4.90), but the difference was not statistically significant. Students who had a positive history of a psychiatric condition had significantly greater loneliness and depression means (M = 20.37, SD = 4.48, and M = 15.79, SD = 6.91) compared to those with a negative psychiatric history (M = 17.40, SD = 4.80 and M = 10.54, SD = 6.15), (*p* < 0.001). Also, students’ monthly family income sufficiency was significantly correlated with loneliness and depression. Students whose monthly family income was enough or enough with savings had significantly lower loneliness mean scores (loneliness: M = 16.78, SD = 4.92 or M = 17.38, SD = 4.40; depression: M = 10.01, SD = 6.10 or M = 10.04, SD = 5.85) compared to those whose families were in debt or had insufficient monthly income (loneliness: M = 19.76, SD = 5.13 or 20.16, SD = 4.30; depression: M = 14.36, SD = 6.36 or M = 15.10, SD = 8.40) (*p* < 0.001). Students with GPA>4 had slightly lower depression means (M = 10.82, SD = 6.27) than whose with GPA ≤ 4 points (M = 11.85, SD = 6.83) (*p* = 0.053).

Regarding COVID-19 related characteristics, students who spent 60 days or more in home quarantine had significantly higher depression mean scores (M = 11.48, SD = 6.55) compared to those who had spent fewer than 60 or 40 days (M = 10.13, SD = 5.77 or M = 9.66, SD = 6.39) (*p* = 0.017). Students who had been diagnosed with COVID-19 disease had significantly lower loneliness, and depression means (M = 15.86, SD = 2.25) than those who had not been diagnosed with COVID-19 (M = 17.74, SD = 4.48) (*p* = 0.009). But students’ family or friend exposure to COVID-19 did not correlate significantly with their loneliness or depression means (*p* = 0.401 or *p* = 0.543).

Multivariate linear regression analysis was used to assess the combined and individual associations between the female students’ sociodemographic and COVID-19 related characteristics and depression with their loneliness mean scores during the COVID-19 pandemic. Table 2 showed that the students’ sociodemographic characteristics, including age, marital state, family monthly income, GPA, and positive history of medical or psychiatric conditions, did not converge significantly on their mean perceived loneliness. However, by considering the other predictor independent variables in the analysis, students at humanities colleges had significantly lower loneliness means compared to all the students at the other colleges combined (beta = −0.647, *p* = 0.016). Also, COVID-19 related characteristics, including days spent in home quarantine and positive diagnosis of COVID-19, did not correlate significantly with the loneliness mean score. Furthermore, the analysis model showed that the depression mean score correlated significantly and positively with the loneliness mean score (beta = 0.419, *p* < 0.001). It is noteworthy that the state of being married predicted a slightly lower loneliness mean, despite the lack of statistically significant mean differences (*p* = 0.153).

**TABLE 2:** Multivariate linear regression analysis of the female students’ perceived loneliness (Riyadh, Saudi Arabia. 2020).

		95% CI beta coefficient		
Variables	Beta coefficient	Lower Bound	Upper Bound	*p*-value
(Constant)	12.756	9.230	16.282	<0.001
Age (years)	0.030	−0.071	0.131	0.555
Married	−0.784	−1.860	0.292	0.153
Family monthly income	−0.125	−0.481	0.231	0.491
Humanities college students	−0.647	−1.173	−0.121	0.016
GPA score	−0.177	−0.652	0.298	0.464
Days spent in home quarantine	−0.220	−0.626	0.186	0.288
Positive history of medical condition	0.230	−0.635	1.095	0.602
Positive history of psychiatric condition	−0.056	−0.928	0.816	0.900
Diagnosed with COVID-19 disease	−1.063	−2.957	0.832	0.271
Depression mean score	0.419	0.367	0.471	<0.001

Dependent variable = Students’ perceived loneliness. Model overall significance: f (11,741) = 60.59, *p* < 0.001. Model R = 68.8, Adjusted R-squared = 46.6%.

## Discussion

To our knowledge, no detailed study had investigated female students’ sense of loneliness and depression in facing the COVID-19 outbreak in Saudi Arabia. This research determines the psychological impact of the outbreak on female students during the 2020 summer vacation. It was found that 63.3% of the female students experienced loneliness and 41.1% experienced depression. And there was a significant positive correlation between loneliness and depression among female students. This can be attributed to the social restrictions calling for physical distancing, which had a negative impact on social activity, networking, and mood [20].

These results were not surprising, as a Saudi national study discovered that the risk of depression increased by 71.2% during the summer of 2020 compared to the pre-pandemic period [9]. Similar results were shown in previous studies conducted in Saudi Arabia during the pandemic. One conducted among people under the Saudi health quarantine noted that the prevalence of depression and anxiety was 49.2% and 44.9%, respectively [21]. Moreover, during the pandemic, moderate to high stress was prevalent (85%) among students studying in virtual classrooms in Saudi Arabia, especially females and university students [11]. Similarly, another two Saudi studies that were conducted during the pandemic showed that university students had significant symptoms of depression (48.8% and 56.3%) and anxiety (40.8% and 40.3%), especially female students [12, 13]. Another study conducted among medical students found that 94% suffered from moderate to high perceived stress, and 47% had anxiety symptoms [22]. Furthermore, an American cross-sectional study showed that loneliness and depression were prevalent among 61.9% and 80%, respectively, of the participants during COVID-19 [23]. In contrast, when comparing our results to some international studies, we noted slight differences. A longitudinal study in Germany comparing the mental health of university students before and during the pandemic found a small increase in depression mean and a medium increase in loneliness [24]. A cross-sectional study conducted in Canada to measure the loneliness among older adults in the community during COVID-19 found that 43.1% of the 4879 participants were lonely during the pandemic [25]. These differences might be due to the difference in the sample size and the age or gender of the participants.

Being married was found to predict a slightly lower level of loneliness in our sample. Marriage has a protective effect on loneliness by building up emotional connectedness [26]. Moreover, this study reported that loneliness and depression were significantly prevalent among female students whose families had insufficient monthly income. This could be explained by the financial cost of virtual socialization and entertainment, including internet bills, websites fees, and electronic devices, which may contribute to social isolation, loneliness, and depression. Also, it was noted that depression is slightly lower among female students with high GPAs, which is consistent with a previous Saudi study among medical students during the pandemic [22]. This can be attributed to academic satisfaction [27, 28]. Additionally, loneliness was reported to be lower among female students in humanities colleges compared to the other colleges. This could be due to the larger number of participants from humanities college (61.5%). It also can be explained by the higher availability of time for socialization compared to other colleges due to less competition or stress.

Furthermore, loneliness and depression were found to be significantly prevalent among female students with a positive history of a psychiatric condition. This finding is in line with previous Saudi studies done during the pandemic among university students [12, 13, 22]. A longitudinal study noted that pre-pandemic depression, distress, and loneliness were predictive factors for depression during the pandemic [24]. This can be explained by the effect of the pandemic in triggering existing psychiatric conditions [29]. This study also noted that loneliness was slightly prevalent among female students with a positive history of a medical condition, which may be due to their strict social isolation to protect themselves from infections. Another Saudi study found that a positive history of a chronic medical condition was a risk factor for depression in university students [12].

Interestingly, the students who had been diagnosed with COVID-19 measured significantly lower loneliness and depression, but having a family or friend with a history of COVID-19 disease didn’t correlate significantly with loneliness or depression. This could be due to social support from the community, which may decrease the sense of loneliness. However, only 14 (1.9%) participants in our study had a positive test of COVID-19, which may limit this finding. Female students’ compliance with home quarantine and the duration spent in quarantine did not correlate significantly with the students’ sense of loneliness. Contrarily, female students who spent 60 days or more in home quarantine had significantly higher depression. Equivalently, a Saudi study done at the beginning of the COVID-19 outbreak stated that depression increased during the later weeks of quarantine [21]. Another Saudi study done during the pandemic found that the duration of quarantine had a notable index of psychological impact [30].

The study had known limitations. First, it had the limitation of convenience sampling, which affects the generalizability of the findings, but we increased the sample size to improve the representation of the sample by recruiting participants from different colleges in the two main universities in the capital city and one of them considered the largest for women in the world. Second, using an online survey might limit our study, but it was a convenient method to reach university students during quarantine. Third, the cross-sectional design of the study does not prove causality relationships and there is a lack of baseline assessment data before the pandemic. Fourth, the sample includes only female students, which limits generalizability to all university students.

In conclusion, loneliness and depression were reported among 63.3% and 41.1% of the female university students in Saudi Arabia over summer vacation during the COVID-19 pandemic and they were correlated positively and significantly. The prevalence of loneliness and depression was significantly higher among students whose families had insufficient monthly income and who had a positive history of a psychiatric condition. This indicates the strong demand for psychosocial interventions for these vulnerable groups during the pandemic. Further longitudinal studies are needed for monitoring and evidence-based interventions.
